# PET imaging of reactive astrocytes in neurological disorders

**DOI:** 10.1007/s00259-021-05640-5

**Published:** 2021-12-07

**Authors:** Yu Liu, Han Jiang, Xiyi Qin, Mei Tian, Hong Zhang

**Affiliations:** 1 Department of Nuclear Medicine and PET Center, The Second Affiliated Hospital of Zhejiang University School of Medicine, 88 Jiefang Road, Hangzhou, 310009 Zhejiang China; 2 Institute of Nuclear Medicine and Molecular Imaging of Zhejiang University, Hangzhou, China; 3 Key Laboratory of Medical Molecular Imaging of Zhejiang Province, Hangzhou, China; 4 PET-CT Center, Fujian Medical University Union Hospital, Fuzhou, 350001 China; 5 College of Biomedical Engineering & Instrument Science, Zhejiang University, Hangzhou, China; 6 Key Laboratory for Biomedical Engineering of Ministry of Education, Zhejiang University, Hangzhou, China

**Keywords:** Positron emission tomography (PET), Reactive astrocytes, Monoamine oxidases-B (MAO-B), Alzheimer’s disease (AD), Parkinson’s disease (PD), Amyotrophic lateral sclerosis (ALS), Multiple sclerosis (MS)

## Abstract

The reactive astrocytes manifest molecular, structural, and functional remodeling in injury, infection, or diseases of the CNS, which play a critical role in the pathological mechanism of neurological diseases. A growing need exists for dependable approach to better characterize the activation of astrocyte in vivo. As an advanced molecular imaging technology, positron emission tomography (PET) has the potential for visualizing biological activities at the cellular levels. In the review, we summarized the PET visualization strategies for reactive astrocytes and discussed the applications of astrocyte PET imaging in neurological diseases. Future studies are needed to pay more attention to the development of specific imaging agents for astrocytes and further improve our exploration of reactive astrocytes in various diseases.

## Introduction

Astrocytes are the most abundant type of glial cells in the brain. The astrocytes are responsible for many basic functions in the central nervous system (CNS). For example, they can provide essential metabolic support for neurons and other cell types, regulate synaptic plasticity, participate in the composition of blood–brain barrier (BBB), and maintain the extracellular ion balance [1]. In recent years, increasing studies have paid attention to the role of astrocytes other than their basal functions, such as participating significantly in the hyperactivity [2] and contributing importantly to remote memory formation [3]. In neurological disorders, the astrocytes undergo significant morphological and molecular phenotypic changes, the most widely characterized of which are cellular hypertrophy and upregulation of the intermediate filament protein GFAP. The hallmark signal of astrocyte reactivity appeared in the early stage of age-related cognitive decline [4]. Unique features of reactive astrocytes are reported in neurological disorders, including Alzheimer’s diseases [5], amyotrophic lateral sclerosis [6], Parkinson’s disease [7], and multiple sclerosis [8]. Thus, there is an urgent need for in vivo visualization strategies for astrocyte activation in various diseases.

Positron emission tomography (PET) is a non-invasive technique that allows the visualization of biologically critical processes at the molecular level in vivo [9]. Therefore, PET has been increasingly applied in neuroimaging research [10, 11]. Utilization of this advanced imaging modality allows for better understand pathological mechanism of astrocytes in neurological diseases, thereby hopefully developing novel strategies for related diseases. Regardless of the fact that imaging of reactive astrocytes is still a young field and in need for development of suitable imaging ligands, some potential radiotracers are still worth our attention.

In the current review, we retrospectively searched the original articles on PET imaging of astrocytes published up to October 2021. The search strategy was to use the following search terms in PubMed: “reactive astrocyte(s),” “positron emission tomography,” “monoamine oxidases-B,” “neurological disorders,” “Alzheimer’s disease,” “Parkinson’s disease,” “Amyotrophic lateral sclerosis,” “Multiple sclerosis” alone and in combination. Only original full papers published in English in international journals with an impact factor were considered. Studies related to the activation of astrocytes, PET imaging agents for astrocytes, and their applications were eligible for inclusion. Each article retrieved was subsequently carefully screened based on the relevance of the abstract, and the reference lists of each study were examined for further relevant publications; 94 literatures ultimately considered relevant were included. Here, we briefly introduced the developments of reactive astrocytes, as well as potential targets for PET tracers to bind, and the application of astrocyte PET imaging in neurological diseases. We also discussed the limitations and challenges for imaging astrocytes in order to promote the development of glial pathology in neurological disease.

## Reactive astrocyte

Reactive astrocyte was first described in the 1970s after discovery of the intermediate filament protein GFAP [12]. Escartin et al. redefined the concept of reactive astrocytes in the latest review [13], and they suggested that the reactive astrocytes are an umbrella term for astrocytes comprising multiple states. Reactive astrocytes refer to the astrocytes undergo remodeling in response to pathology, which involves a series of changes from transcriptome to pathology, eventually leading to the imbalance and disturbances of neural homeostasis. Therefore, reactive astrocytes refer to the astrocytes in pathological state, excluding the plasticity regulation of physiological astrocytes.

GFAP served as the most widely used marker of reactive astrocytes, whose significance has also been questioned. The extent of GFAP upregulation in reactive astrocytes generally correlates with the severity of injury, but the correlation is not always proportional, perhaps due to regional differences in astrocytes, including differences in basal GFAP content [14, 15]. The astrocytes in some specific regions, such as the hippocampus, are more GFAP abundant than those in cortex, thalamus, and striatum of the healthy mouse brain. However, it does not mean that astrocytes in the hippocampus are more reactive, but rather regionally heterogeneous. Besides, the expression of GFAP would naturally change in response to physiological activities [16, 17]. Thus, although the protein expression of GFAP is commonly used to evaluate the reactivity of astrocytes in injury and disease, better astrocyte markers are still needed. Other astrocyte markers such as aldolase-C (ALDOC), glutamine synthetase (GS), and aldehyde dehydrogenase-1 L1 (ALDH1L1) should be used together to correctly estimate the astrocytes. However, immunohistochemical staining for these cytosolic enzymes still fail to reveal detailed processes.

Meanwhile, transcriptomics provides more marker genes to rigorously identify reactive astrocytes. The gene analysis of reactive astrocytes in mice found that neuroinflammation and ischemia induced two distinct types of astrocytes, termed “A1” and “A2” [18]. A1 neuroinflammatory astrocytes upregulate genes that have previously been proved to be synaptic destructive (such as complement cascade genes), suggesting that A1s may have a “harmful” function. Comparatively, ischemia-induced A2 astrocytes upregulate neurotrophic factors which can promote neuronal growth and development, suggesting that A2s may have “beneficial” functions. Subsequent studies appeared to confirm this hypothesis. For example, RNA sequencing of astrocytes found that senescent astrocytes present a reactive phenotype of neuroinflammatory A1-like reactive astrocytes [19]. Moreover, the type A1 astrocytes had activation of NF-κB/C3/C3aR signaling pathway which may contribute to synaptic dysfunction in AD [20], while the formation of A2 astrocytes scar contributes to CNS axon regeneration [21]. Afterwards, some studies tend to use A1- and A2-specific markers (such as C3 and S100a10) to label the phenotype of astrocytes [22–24]. However, the simple dichotomy may also not reflect the heterogeneity of astrocytes in the brain [25–27]. Hasel et al. used a multimodal approach including RNA sequencing, spatial transcriptomics, gene map, and dataset integration to profile the response of astrocytes to inflammatory [28]. It is found that different subsets of astrocytes have spatial, temporal, gender, and subtype-specific responses to inflammation, which provides an important reference for the evolution of heterogeneous responses of astrocytes. Consequently, future studies need to cautiously explain the response between different subsets of astrocytes in different diseases, rather than regard the change of astrocytes as a unitary and stable process.

## PET tracers for reactive astrocytes

**Fig. 1:**
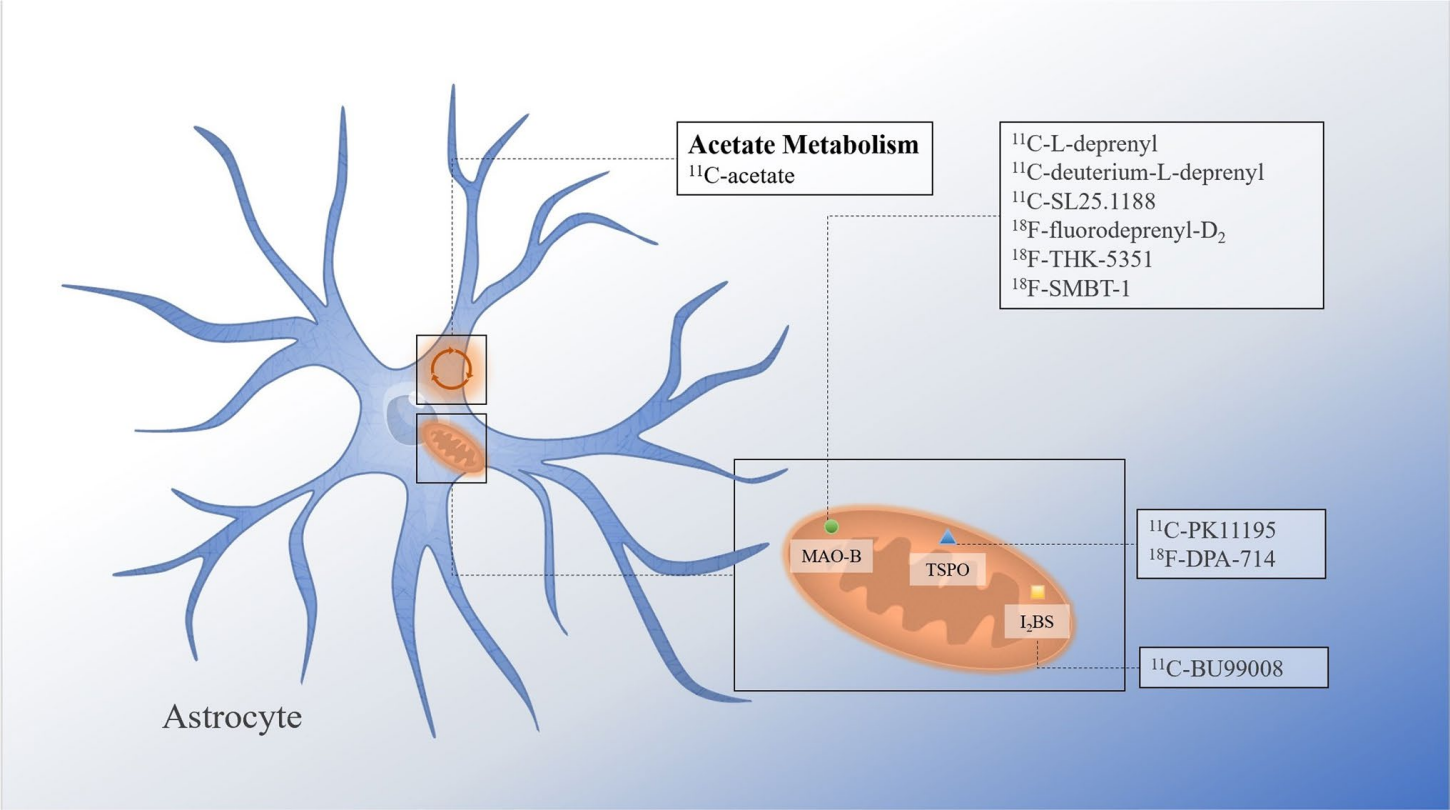
Illustration of tracer bind sites on astrocyte

As pharmaceutical work begins to focus on astrocyte-specific targets, some neuroimaging tools are increasingly needed to visually assess and monitor astrocyte activation in vivo, so as to determine the promising responder to cell-specific drugs and carry out corresponding clinical transformation [29]. The development of PET tracers for astrocytes makes the visualization of reactive astrocytes possible. PET tracer binding sites for astrocytes are shown in Fig. 1. In the section, we will introduce PET radiotracers that have been developed for astrocyte.

### MAOB-based radiotracer

Monoamine oxidases (MAO), first discovered in the liver by Mary Bernheim [30], have two subtypes, MAO-A and MAO-B. MAO-B is the main subtype in the human brain, accounting for about 70% of the total brain MAO activity [31, 32]. MAO-B, a major enzyme that metabolizes dopamine and histamine, is highly expressed in the outer mitochondrial membrane of astrocytes in the human brain, which is considered one of the PET imaging markers of astrocytes. So far, many different radiotracers have been developed for imaging MAO-B in the reactive astrocytes.

#### Irreversible inhibitors

The first MAO-B radiotracer to be used for PET imaging in human was ^11^C-L-deprenyl [33]. Stereoselectivity of MAO-B for ^11^C-L-deprenyl was demonstrated by comparison of brain uptake and retention of the ^11^C-labeled inactive (D-) and active (L-) enantiomers. The study showed in the brain region rich in MAO-B, the inactive enantiomers were cleared rapidly and the active enantiomers were retained [34]. Therefore, new strategies are needed to improve the pharmacokinetics of imaging agents. In order to slow down the capture rate, deuterated analogues were prepared by using the kinetic isotope effect (KIE). To that end, deuterium-labeled isotopologues ^11^C-deuterium-L-deprenyl (^11^C-DED) has been successfully applied to human PET research [33]. ^11^C-DED has also been widely used and applied to examine the changes of MAO-B in neurodegenerative diseases.

Irreversible inhibitors have great limitations in clinical applications due to the properties of irreversible binding, which include (i) the quantification of binding difficult because of rapid occurrence of irreversible binding and (ii) the formation of impermeable radioactive drugs which may be harmful to the brain. Since these limitations not only confuse image analysis, but also affect clinical transformation to a certain extent, other types of imaging agents, such as reversibly bound imaging agents, have also begun to be prepared and developed.

#### Reversible inhibitors

^11^C-SL25.1188, a reversible imaging agent used in the human brain, shows favorable characteristics in preclinical studies of MAO-B imaging of non-human primate (NHP) brain [35]. In the development of synthesizing this reversible inhibitor, the difficulty is to overcome the technical challenge of ^11^C-CoCl_2_ preparation. Due to the strict requirements of experimental environment and technology, it can only be synthesized in a few laboratories in the world. Obviously, the clinical application of reversible imaging agents was greatly limited. In order to overcome this limitation, some scientists directly carry out intramolecular cyclization from ^11^C-CO_2_ through automatic one-pot procedure, which avoids the use of CoCl_2_ and optimizes the synthesis of reversible imaging agents. Reversible imaging agents have distinct advantages in comparison with irreversible imaging agents. Higher brain uptake and lower metabolic rate greatly improve the quality of PET imaging, and the reversible combination also ensures the biosafety of agents. In the research of the human brain, dynamic modeling using high-resolution PET showed the high uptake of ^11^C-SL25.1188, which was significantly correlated with MAO-B levels of postmortem measurement in the brain [33]. Thus, ^11^C-SL25.1188 have great prospects for radioactive tracing of MAO-B in neurological studies.

However, the short half-life of ^11^C limits the clinical application of ^11^C-labled imaging agent due to the requirement of on-site cyclotron. Therefore, ^18^F-labeled imaging agent has been developed for MAO-B imaging because of its longer half-life [36, 37]. Sangram Nag et al. have made many efforts to the development of ^18^F-labeled MAO-B radioligand for years. They developed three new fluorine-18 analogues of L-deprenyl in the early years [38]. Later, they synthesized ^18^F-fluorodeprenyl-D_2_ which has faster kinetics and higher sensitivity than ^11^C-D_2_-deprenyl [36]. Besides, ^18^F-THK-5351 was originally designed to detect neurofibrillary tangles in vivo, and was found to bind MAO-B with high affinity [39–42]. The non-selective binding of THK-5351 to MAO-B and tau limits its clinical utility as a biomarker. In recent study, ^18^F-SMBT-1 was successfully developed through lead optimization from first-generation tau PET tracer ^18^F-THK-5351 [37]. In vitro autoradiography of ^18^F-SMBT-1 using human brain tissues, SMBT-1 showed the highest affinity for MAO-B and an excellent binding selectivity for MAO-B over MAO-A, amyloid, and tau aggregates. Because of excellent reversible binding characteristics, ^18^F-SMBT-1 was allowed to perform repeated PET scans in the same subject, which would be helpful for longitudinal quantification of astrogliosis. Therefore, ^18^F-SMBT-1 may be a promising candidate as a selective and reversible imaging tracer for MAO-B.

### Non-MAOB-based radiotracer

Imidazoline_2_ binding sites (I_2_BS) are another reliable marker to specifically visualize astrocyte activation in the human brain under physiological and pathological conditions. I_2_BS is mainly expressed in the outer mitochondrial membrane of astrocytes, but much less in neurons [43]. Importantly, changes in I_2_BS are associated with increased astrocyte activation in a series of diseases [44]. Tyacke et al. have synthetized a new PET-tracer called ^11^C-BU99008, specifically targeting I_2_BS [45]. A recent study has shown that ^11^C-BU99008 could detect I_2_BS expressed by reactive astrocytes with fine selectivity and specificity, and visualize reactive astrogliosis in postmortem AD brains [46], hence be a potential clinical astrocytic PET tracer to further understand the role of reactive astrocyte in AD.

In addition, the application of PET tracers targeting TSPO in astrocytes is quite limited, such as ^11^C-PK11195 and ^18^F-DPA-714. Previous studies considered TSPO as a marker of microglia, but in recent years, more and more research have focused on the cell source of TSPO. Significantly, a study of the cellular origin of TSPO signaling in AD found that the early signal of TSPO came from astrocytes, while in the later stage, it was mainly microglia [47]. The results indicated that the interpretation of TSPO imaging depends on pathological stage, and emphasizes the special role of astrocytes. In another study on the source of TSPO cells, the researchers found that in neurotoxicant-induced animal model of demyelination, TSPO signal was initially provided by microglia, while in a later stage (such as 6 weeks later), the signal source of TSPO was mainly from astrocytes [48]. These studies remind us that the signal source of TSPO is not only composed of microglia, but also astrocytes. With the understanding of disease pathology, according to the different stages of disease development, TSPO may provide us with some astrocyte information [49].

## PET imaging of reactive astrocytes in neurological diseases

A large number of studies have noticed that the pathological changes of astrocyte may be an important link in the pathogenic cascade of neurological diseases. There is no doubt that the visualization of astrocytes by PET imaging can improve our better understanding of glial pathological changes. Here, we introduced four diseases with astrocyte PET imaging applications, and discussed the significance of corresponding PET signal changes. In addition, some neurological disorders related to astrocyte pathology but without specific PET research are also mentioned in the last part.

### Alzheimer’s disease

**Fig. 2:**
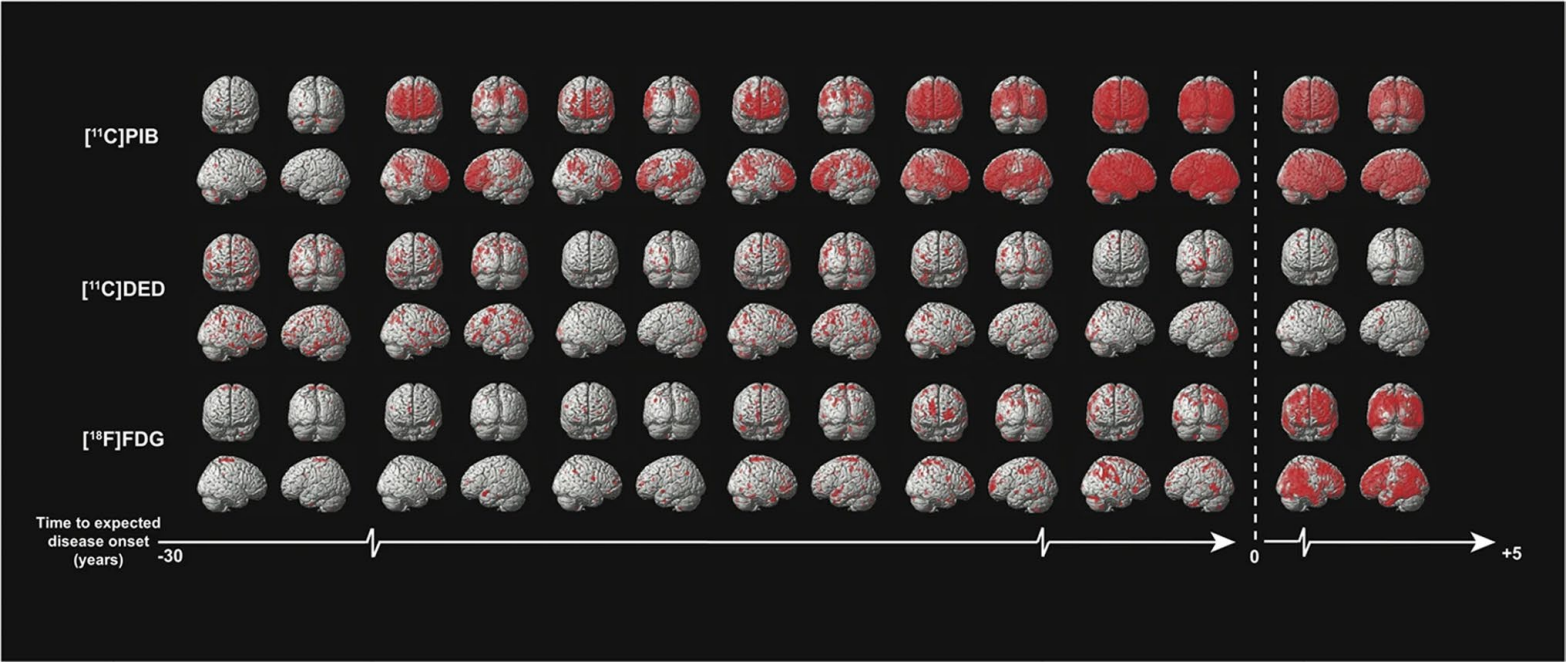
Progression of PET biomarkers in ADAD mutation carriers. Compared with five age-matched non-carriers, each pair of columns represents a single mutation carrier. Each pair of rows represents a different PET biomarker. The scale from left to right indicates the approximate time (in years) that clinical symptoms are expected to appear. (Reprint permission was obtained from reference [50])

Alzheimer’s disease (AD) is a progressive neurodegenerative disorder with gradually cognitive decline [50]. In addition to the well-known neurofibrillary tangles and Aβ deposition, glial activation and neuroinflammation also drive the progression of AD [4, 51, 52]. However, most PET imaging studies of neuroinflammation have focused on microglia, and there are few PET tracers of reactive astrocytes [53, 54]. The ^11^C-deuterium-L-deprenyl (^11^C-DED) has been widely used to detect activated astrocytes in patients with AD, and it can simultaneously measure cerebral perfusion and astrocytes in a single PET scan, thereby providing both functional and pathological information [55]. In a study using ^11^C-DED for PET imaging, the cerebellar gray matter was selected as a reference, and the ^11^C-DED data of healthy people (*n* = 14), MCI patients (*n* = 8), and AD patients (*n* = 7) were dynamically analyzed. The results showed that increased ^11^C-DED uptake occurs early in the development of AD, especially in mild cognitive impairment (MCI) ^11^C-PIB^+^ patients’ brains [56]. The same results were observed in autosomal dominant Alzheimer’s disease (ADAD). Compared with the imaging results of ^18^F-FDG and ^11^C-PIB, ^11^C-DED images showed a prominent increase in MAO-B in the early stage of clinical symptom onset (Fig. 2), which strengthens the possibility the activation of astrocytes may be an early driving force of Alzheimer’s disease pathology [50]. In PIB^+^ MCI patients, the parahippocampal astrocytosis showed by PET was associated with decreased GM density measured by MRI [57], which may suggest that the increase of parahippocampal astrocytes may affect the loss of gray matter cells in patients with prodromal Alzheimer’s disease. However, in patients with Alzheimer’s disease, the increase of astrocytes is less related to the loss of brain cells, but more related to the amyloid process. Subsequent preclinical studies demonstrated that compared with the APPswe mice at 8–15 months and 18–24 months, the binding of ^11^C-DED in cortex and hippocampus of 6-month-old APPswe mice was twice as much as the former two. Longitudinally, ^11^C-DED binding decreased with age in APPswe mice, but did not change significantly in WT mice. Notably, immunostaining results showed scarce diffuse Aβ_42_ deposits in the cortex and hippocampus of 6-month-old APPswe mice, whereas obvious increases were observed in 18—to 24-month-old APPswe mice, which may indicate that astrocytosis occurs before the deposition of Aβ plaques [58]. In addition, the PET study using imidazole receptor imaging agents showed that an increase in ^11^C-BU99008 uptake in cognitively impaired (CI) patients compared with healthy controls (HC) [59], and the autoradiography showed that ^3^H-BU99008 was colocalized with GFAP staining, which confirmed the results of in vivo PET imaging. These findings are consistent with the results of postmortem [60] and preclinical studies [61]. MAO-B off target binding brought ^18^F-THK5351 into disrepute in the past few years. ^18^F-THK5351, initially considered as a tau imaging agent, was later found to have a certain non-specific binding with MAO-B. With the development of glial pathology, ^18^F-THK5351 provides great value for the diagnosis of AD and the classification of MCI because of which can simultaneously evaluate the pathological progress of tau and reactive astrocytes [62]. The information provided by PET imaging seems to be consistent with the discovery of other liquid biomarkers of reactive astrocytes in AD. For example, many recent studies have confirmed that the level of GFAP in plasma and CSF increases in the preclinical and symptomatic stages of AD [63–66]. In addition, CSF YKL-40, as a biomarker of reactive astrocyte subsets, also increased in preclinical AD patients [67, 68]. In general, these studies have proved astrocyte activation is an early pathological event in the development of AD. Future studies could combine specific astrocyte PET imaging with liquid biomarker data to better explain a series of behaviors of astrocytes in AD.

### Parkinson’s disease

**Fig. 3:**
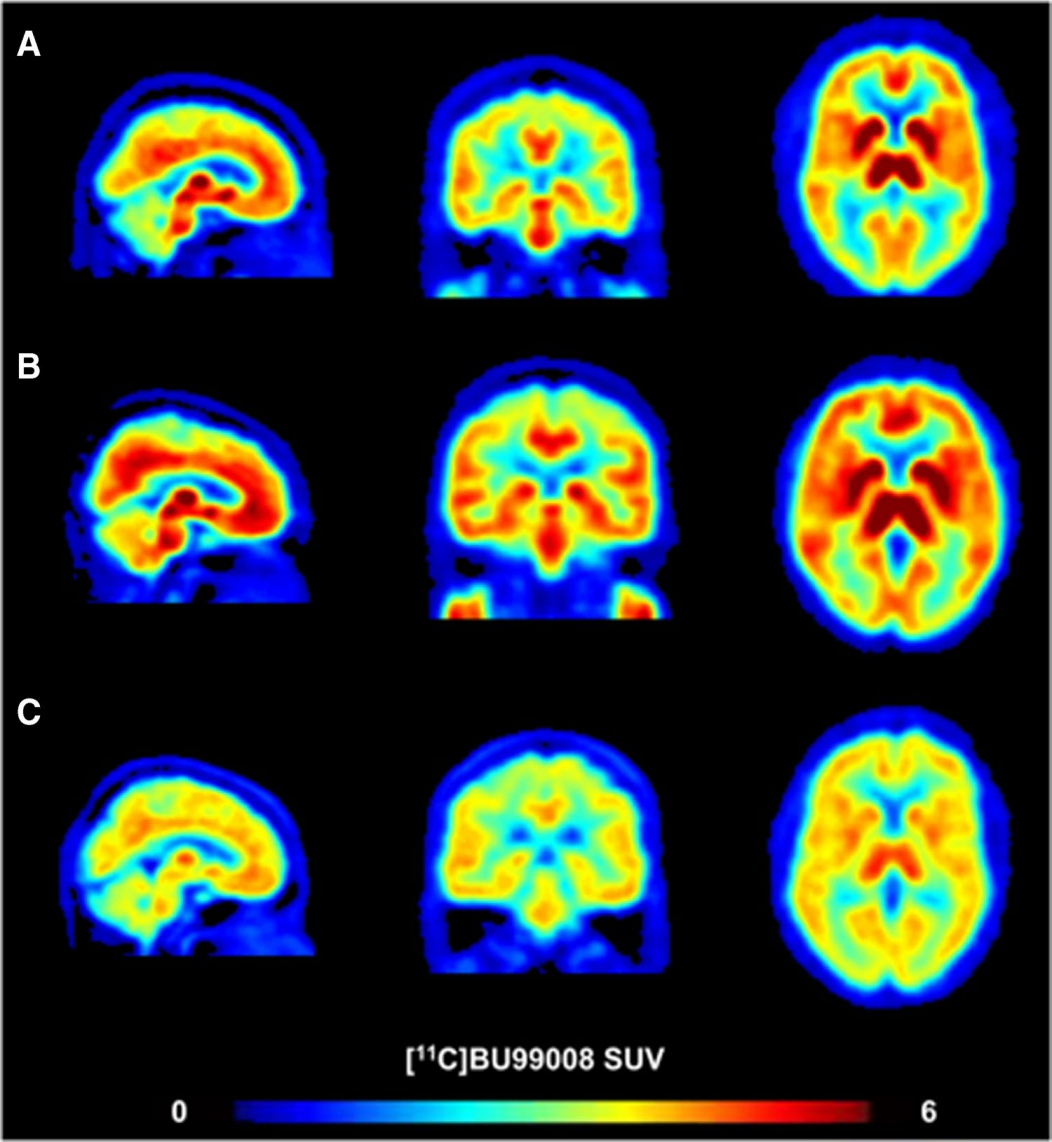
^11^C-BU99008 standardized uptake value. Compared with the healthy control (**A** ; 65-year-old male), early Parkinson’s disease patients (**B**; 60-year-old female; disease duration 2 years) demonstrated more ^11^C-BU99008 uptake, which represented increased I_2_BS expression, whereas moderate/advanced Parkinson’s disease patients (**C**; 63-year-old male; disease duration 16 years) showed a global loss of it. (Reprint permission was obtained from reference [7])

Parkinson’s disease (PD) is a common dyskinesia disorder. The pathological features of PD mainly include loss of substantia nigra striatum and accumulation of α-synuclein [69]. With astroglia α-synuclein-positive cytoplasmic accumulations have been shown post-mortem in patients with PD, increasing evidences suggest that reactive astrocytes may take part in the pathophysiology of PD [70, 71]. ^11^C-BU99008 PET can indirectly evaluate the activation of astrocytes by measuring the expression of I_2_BS [72, 73]. A study using ^11^C-BU99008 PET molecular imaging showed increased expression of I_2_BS in the cortex and brain stem in patients with early Parkinson’s disease. Compared with the healthy control group, ^11^C-BU99008 V_T_ in the brain stem of patients with early Parkinson’s disease increased by about 52%. Conversely, in patients with moderate and advanced Parkinson’s disease, the expression of I_2_BS decreased in the cortex and subcortical regions (Fig. 3), reflecting the loss of astrocyte function as the disease progressed [7]. Nevertheless, the reports on astrogliosis in Parkinson’s disease are conflicting. With MAO-B be used as a biochemical imaging marker for astrocyte activation in the human brain, a post-mortem study on parkinsonian dopamine deficiency disorders showed both astrocyte markers (GFAP, vimentin, Hsp27) and MAO-B levels were increased and significantly correlated in the putamen of patients with multiple system atrophy (MSA); the same results were observed in the substantia nigra of patients with progressive superanuclear palsy (PSP). However, in the substantia nigra of patients with PD, the levels of MAO-B were normal [74, 75]. Findings from a study using ^18^F-THK5351 imaging in parkinsonian syndromes are consistent with previous postmortem studies [76], which implies that the application of MAO-B-targeted PET imaging in Parkinson’s disease requires a more cautious interpretation. Taken together, correct diagnosis remains a significant clinical challenge due to overlapping signs and symptoms of Parkinson’s syndrome. In combination with biomarkers related to pathological changes and clinical phenotypes, PET imaging of reactive astrocytes is a limited but promising way to evaluate disease progression and drug treatment efficacy.

### Amyotrophic lateral sclerosis

Amyotrophic lateral sclerosis (ALS), the third largest neurodegenerative disease after AD and PD, is a progressive neurodegenerative disease characterized by death of motor neurons in the cerebral cortex and/or brainstem and spinal cord [77]. Increasing evidences suggested that non-neuronal cells play an important role in the pathogenesis of ALS, especially astrocytes in glial cells [77, 78]. Reactive astrocytes have been described in postmortem central nervous system tissues of patients with ALS [79–81]. The transcriptional profile of astrocytes in ALS was changed [82] and reactive astrocytes in ALS display diminished intron retention [6]. The recent study has shown that reactive astrocyte activator knockout can slow down the disease progression of ALS mouse model [22].

**Fig. 4:**
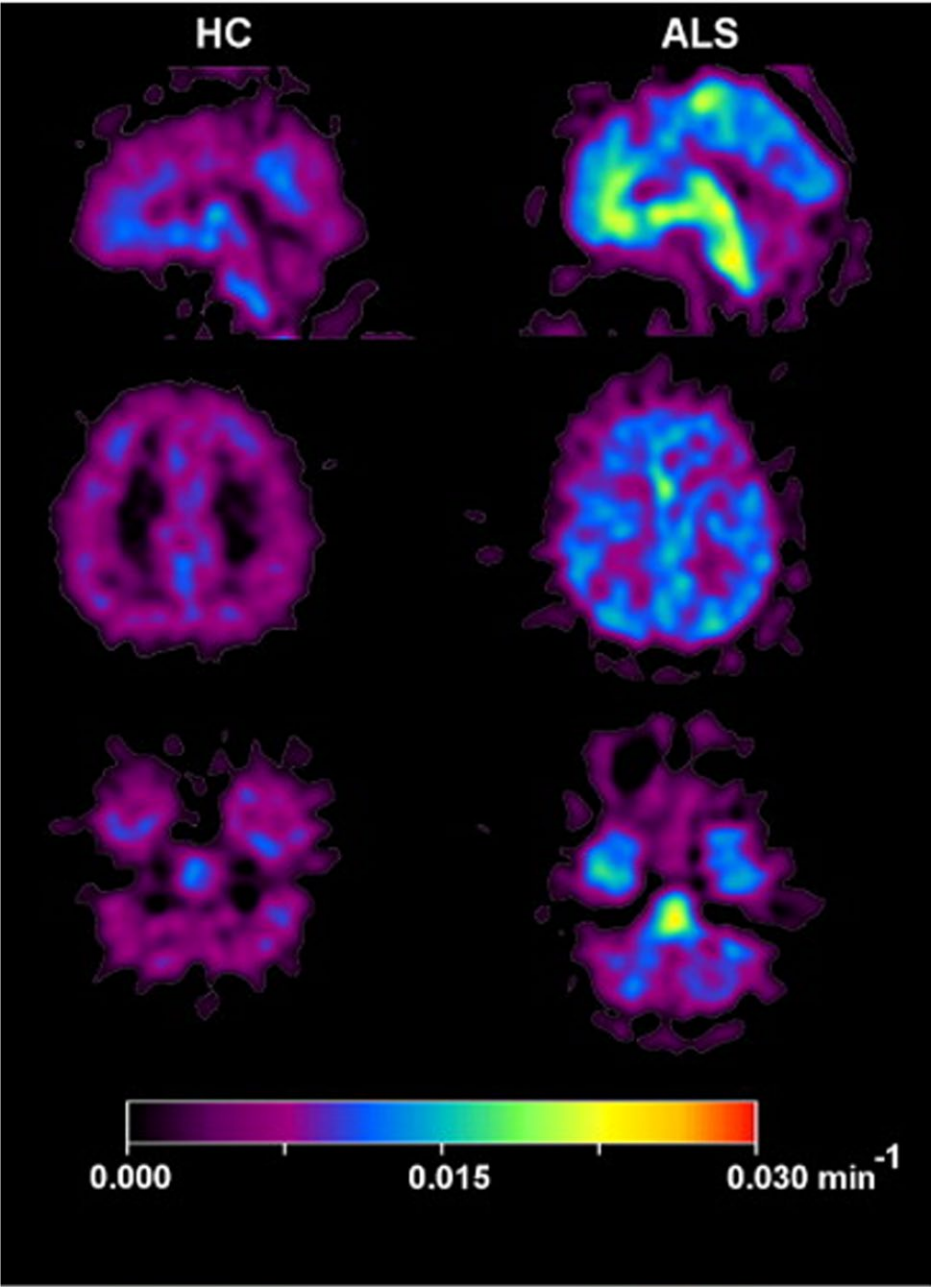
^11^C-DED PET parametric images of ALS patient and healthy control. Compared with the healthy control, the uptake rates of pons and white matter in ALS patients were significantly increased. (Reprint permission was obtained from reference [83])

PET molecular imaging evidence showed that ^11^C-DED the binding rate increased in pons and white matter in ALS patients [83] (Fig. 4). In this study, seven patients with ALS were included and their clinical characteristics were summarized and seven healthy subjects were included as controls. All subjects were studied with ^11^C-DED. The results showed that in addition to the increased combination of white matter and pons, the SUV in the parietal cortex (3.5 ± 0.45) and temporal cortex (3.7 ± 0.32) of patients with ALS was significantly lower than that in the control group (parietal cortex: 4.2 ± 0.38; temporal cortex: 4.2 ± 0.28), which may reflect the decrease of cerebral blood flow in these regions in ALS patients. The increase of white matter binding rate is consistent with other neuropathological evidences [84], but the increase of pontine binding rate may need to be explained more carefully in the absence of other evidence. The high background activity of MAO-B and the small sample size of this study limit the correlation analysis between its imaging manifestations and clinical symptoms. Two of the patients who subsequently underwent the second PET imaging had the same imaging findings as the previous one, but their clinical progress deteriorated, which is a frustrating fact that inhibited the enthusiasm of ^11^C-DED as an alternative marker. Using the reference value of cerebellum for standardization, ^18^F-THK5351 PET scanned images showed increased uptake of medial motor cortex and prefrontal lobe in two different phenotypes of ALS patients. The imaging lesions were consistent with their respective neurological manifestations, but the case lacked specific quantitative analysis and other related evidences [85]. Therefore, the application of astrocyte PET imaging in ALS is still relatively limited and requires more comprehensive exploration.

### Multiple sclerosis

**Fig. 5:**
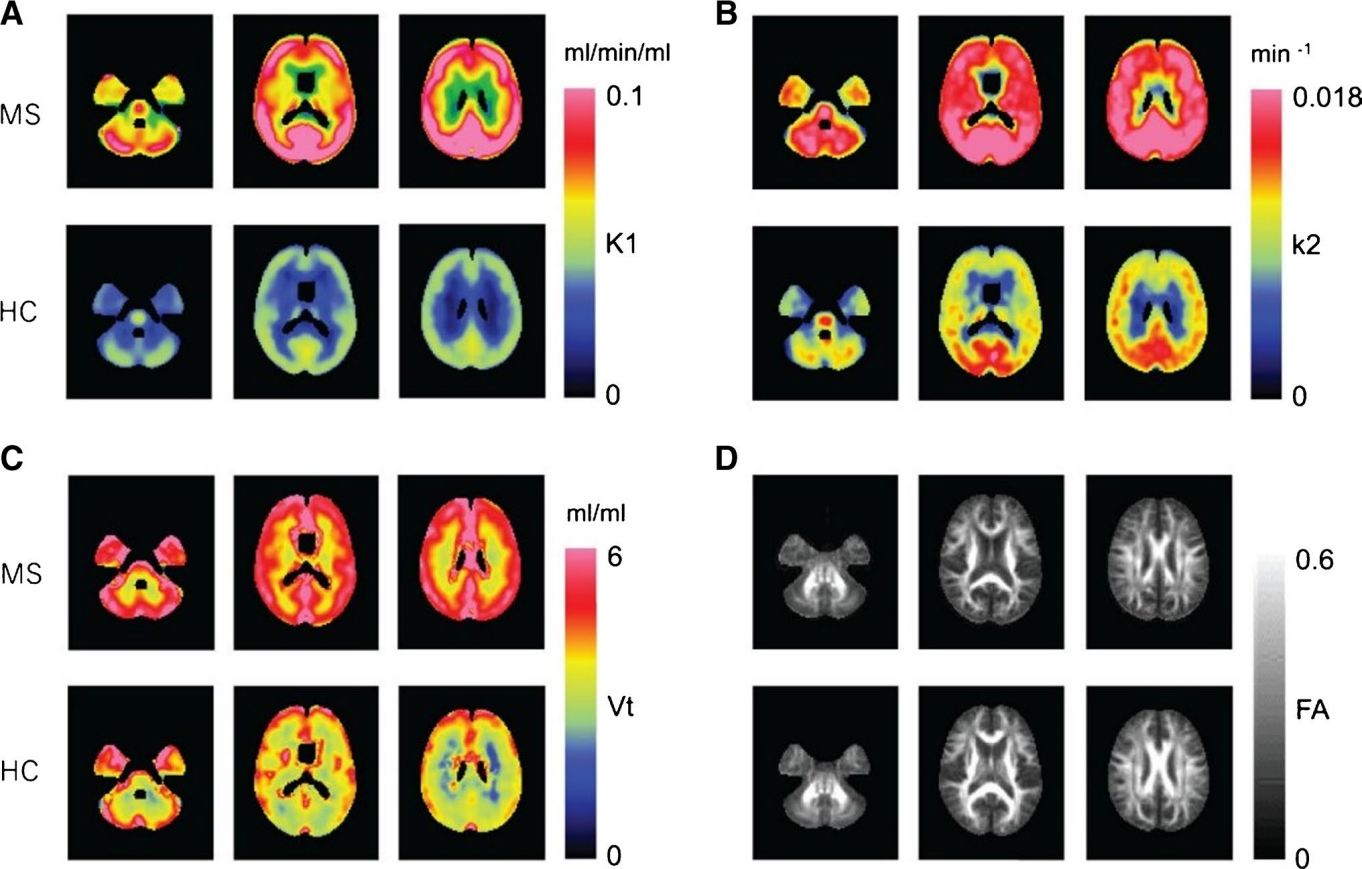
Anatomically normalized group mean images of 1-C-11 acetate in the MS and NC groups. k1 (**a** ) represents influx of acetate; k2 (**b**) represents clearance of total radioactive molecules generated from acetate metabolism in the brain; Vt (**c**) represents radioactivity distribution volume; FA (**d**) represents the fractional anisotropy. (Reprint permission was obtained from reference [93

Multiple sclerosis (MS), a chronic inflammatory demyelinating disease of the CNS, is the most common disabling neurologic disease of young people [86]. According to the clinical characteristics, MS is divided into three different courses: relapsing–remitting (RR), secondary progressive (SP), and primary progressive (PP). The most common is relapsing remitting MS (RRMS), which is characterized by spontaneous regression of neurological dysfunction [87]. Astrocyte reactivity plays an important role in the pathological process of MS, which widely exists from the early stage of lesion formation to the late chronic stage [88]. In multiple sclerosis, astrocytes show a highly reactive phenotype with large cell bodies and processes; they also recruit lymphocytes, cause immune activation, and promote the development of acute lesions. In addition, the foot process of astrocytes is destroyed, which leads to the damage and dysfunction of blood–brain barrier. As the disease turns chronic, the GFAP filaments of astrocytes become accumulated and participate in the formation of glial scar in the later stage of astrocytes [89]. One strategy for visualization of astrocytes is ^18^F-THK5351 PET imaging. With the superimposition of the ^18^F-THK5351 image on the fluid attenuation inversion recovery, one study confirmed that the lesions accumulated by ^18^F-THK5351 structurally correspond to multiple sclerosis plaques [39]. Another strategy for astrocyte imaging is to measure ^11^C-acetate, which is mainly absorbed and metabolized in brain astrocytes [90, 91]. In the previous study, ^11^C-acetate PET was used to study the imaging of MS patients; 6 RRMS patients in remission and 6 healthy controls were recruited. The average SUV in each subject’s bilateral thalami was used as a reference to calculate the relative standardized uptake value (SUVt) [92]. The results showed that the average SUV of MS patients was higher than that of healthy subjects in all evaluation regions, especially in the parietal lobe, occipital, and insular regions. The main drawback of this study is that it only includes the analysis of PET static data. Another method using kinetic parameters was used for the determination of ^11^C-acetate, which used k2 value (total clearance of acetate metabolism in the brain) to reflect the metabolic rate of acetate in astrocytes [93]. The results showed that the white matter/gray matter ratio k2 in MS patients was significantly higher than that in normal subjects (Fig. 5), and the most obvious change in k2 occurred in neuronal fiber bundles, which means that astrocytes near myelin sheath or axon are mainly activated in MS. However, there are only 8 patients in this study and they are not homogeneous. It is impossible to determine the effect of disease subtypes on astrocyte metabolism, and the contribution from other non-astrocytes in k2 remains to be clarified. Therefore, further research is still needed to improve the accuracy of measurement. Nevertheless, there are few studies on astrocyte imaging and further neuroimaging studies are needed to visually explore the role of astrocytes in MS.

### Other neurological disorders

The proliferation of glial cells after cerebral ischemia leads to neuroinflammation and further injury and neuronal death. It is noteworthy that the expression of astrocytes is different from that in the neurodegenerative diseases mentioned above. In the neuroinflammation caused by cerebral ischemic stroke, the activation of microglia and the infiltration of macrophages first appeared, and then caused the response of astrocytes [94]. The reactive astrocytes are observed in the surrounding area of infarction. In addition to releasing some proinflammatory factors, astrocytes can also form glial scars to limit the spread of inflammation [95]. The molecular basis of astrocytes between harmful and beneficial needs more evidence to be analyzed. Transcriptomic evidences supported the importance of astrocyte in neuroprotection after stroke. Rakers et al. identified the differential expression of 1003 genes and 38 transcription factors after experimental ischemic stroke [96]. The results showed that the upregulation of A2 neuroprotective gene was more advantageous than that of A1 gene. This also confirms the conclusion of a previous study [97]. The extensive application of TSPO-PET in neuroinflammatory imaging provides evidences for the role of astrocytes in cerebral ischemia [48]. Some studies suggest that the cell source of TSPO signal after injury comes from microglia at early stage (2w), and later (6w) from astrocytes [98, 99]. The conclusion refers to the results of immunostaining, but the argument is not sufficient. The application of more specific imaging agents targeting astrocytes can obviously provide more explanations for reactive astrocytes in ischemic injury, but the current research is rarely focused on this aspect.

About 80% of optic neuromyelitis is closely related to the autoantibody of astrocyte aquaporin-4 (AQP4) [100]. In AQP4-IgG-positive optic neuromyelitis, the loss and dysfunction of astrocytes lead to the secondary loss of oligodendrocytes and neurons [101]. In the relevant case reports, more cases used ^18^F-FDG-PET to represent the astrocyte injury and hypermetabolic state of the disease [102, 103], which is not specific enough to reflect the pathological characteristics. The same examples, such as autoimmune GFAP astrocytopathy [104] and Alexandria disease [105], are caused by astrocyte changes. The application of PET molecular imaging targeting astrocyte-specific proteins can greatly improve our knowledge of progress in these diseases.

## Conclusions and future directions

PET imaging of astrocytes has contributed to our better understanding of their pathological evolution in disease and to the development of astrocyte-related intervention strategies. Evidences from PET imaging indicated the activation of astrocytes appears at an early stage in AD. While in PD, PET imaging of activated astrocytes needs to select more suitable targets rather than MAO-B. In ALS and MS, the regions with abnormal PET signal correlated with features of the diseases, which may suggest that astrocyte PET imaging is a promising technique but requires further exploration.

PET imaging of reactive astrocytes is still in an immature stage. Firstly, the multitude of complex subtypes of astrocytes dictates the need for multiple molecular imaging markers such as enzymes, receptors, or metabolic markers to delineate their specific states, and the development of ligand imaging agents for these targets is now still very limited. Moreover, the specificity remains to be improved for imaging agents that have been applied to astrocyte imaging, and current applications need to incorporate more additional data to better interpret imaging signals. Besides, the choice of reference region in astrocyte imaging was not fixed and tended to select the brain region with the least difference from healthy controls in these studies (Table 1), which may consider that the affected brain regions of astrocyte activation have different distributions in different diseases. Hence, the imaging of astrocytes needs to be combined with the clinical diagnosis to determine suitable reference region.

**Table 1:** Reference regions of different radiotracers for astrocyte imaging

Disease	Radiotracer	Targets	Sample	Reference region	Reference
AD	^11^C-DED	MAO-B	29	Cerebellar gray matter	[56]
	^11^C-BU99008	I_2_BS	20	Cerebellar gray matter	[59]
	^18^F-THK-5351	MAO-B, tau	97	Cerebellar gray matter	[62]
PD	^11^C-BU99008	I_2_BS	36	None	[7]
	^18^F-THK-5351	MAO-B, tau	34	Global mean	[76]
ALS	^11^C-DED	MAO-B	14	Occipital cortex	[83]
MS	^11^C-acetate	MAO-B	12	Bilateral thalami	[92]

*AD*, Alzheimer’s disease; *PD*, Parkinson’s disease; *ALS*, amyotrophic lateral sclerosis; *MS*, multiple sclerosis; *MAO-B*, monoamine oxidases-B; *I*_*2*_*BS*, imidazoline2 binding sites

A key step forward in the field would be the development of imaging agents that specifically target astrocyte subpopulations. These ideal candidate agents should have characteristics of relatively long half-life, eminent binding specificity, and high affinity, the most promising of which are screened and applied in patients’ cohorts with different stages of neurological disorders. Use this as a foundation, future studies will enable real-time longitudinal detection of astrocyte pathological progression in various diseases, thereby facilitating our better understanding of the sequence and time of astrocyte pathology in neurological diseases, and contributing to developing successful intervention strategies for the regulation of astrocytes.

## References

[1] Verkhratsky A, Nedergaard M (2018). Physiology of astroglia. Physiol Rev.

[2] Nagai J, Rajbhandari AK, Gangwani MR, Hachisuka A, Coppola G, Masmanidis SC (2019). Hyperactivity with disrupted attention by activation of an astrocyte synaptogenic cue. Cell.

[3] Kol A, Adamsky A, Groysman M, Kreisel T, London M, Goshen I (2020). Astrocytes contribute to remote memory formation by modulating hippocampal-cortical communication during learning. Nat Neurosci.

[4] Price BR, Johnson LA, Norris CM (2021). Reactive astrocytes: the nexus of pathological and clinical hallmarks of Alzheimer’s disease. Ageing Res Rev.

[5] Lopes CR, Cunha RA, Agostinho P (2021). Astrocytes and adenosine A receptors: active players in Alzheimer’s disease. Front Neurosci.

[6] Ziff OJ, Taha DM, Crerar H, Clarke BE, Chakrabarti AM, Kelly G (2021). Reactive astrocytes in ALS display diminished intron retention. Nucleic Acids Res.

[7] Wilson H, Dervenoulas G, Pagano G, Tyacke RJ, Polychronis S, Myers J (2019). Imidazoline 2 binding sites reflecting astroglia pathology in Parkinson’s disease: an in vivo11C-BU99008 PET study. Brain.

[8] Schirmer L, Velmeshev D, Holmqvist S, Kaufmann M, Werneburg S, Jung D (2019). Neuronal vulnerability and multilineage diversity in multiple sclerosis. Nature.

[9] Tian M, He X, Jin C, He X, Wu S, Zhou R (2021). Transpathology: molecular imaging-based pathology. Eur J Nucl Med Mol Imaging.

[10] Zhang K, Mizuma H, Zhang X, Takahashi K, Jin C, Song F (2021). PET imaging of neural activity, beta-amyloid, and tau in normal brain aging. Eur J Nucl Med Mol Imaging.

[11] Hansson O (2021). Biomarkers for neurodegenerative diseases. Nat Med.

[12] Eng LF, Vanderhaeghen JJ, Bignami A, Gerstl B (1971). An acidic protein isolated from fibrous astrocytes. Brain Res.

[13] Escartin C, Galea E, Lakatos A, O’Callaghan JP, Petzold GC, Serrano-Pozo A (2021). Reactive astrocyte nomenclature, definitions, and future directions. Nat Neurosci.

[14] Escartin C, Guillemaud O, Carrillo-de Sauvage M-A (2019). Questions and (some) answers on reactive astrocytes. Glia.

[15] Ben Haim L, Rowitch DH (2017). Functional diversity of astrocytes in neural circuit regulation. Nat Rev Neurosci.

[16] Rodríguez JJ, Terzieva S, Olabarria M, Lanza RG, Verkhratsky A (2013). Enriched environment and physical activity reverse astrogliodegeneration in the hippocampus of AD transgenic mice. Cell Death Dis.

[17] Gerics B, Szalay F, Hajós F (2006). Glial fibrillary acidic protein immunoreactivity in the rat suprachiasmatic nucleus: circadian changes and their seasonal dependence. J Anat.

[18] Zamanian JL, Xu L, Foo LC, Nouri N, Zhou L, Giffard RG (2012). Genomic analysis of reactive astrogliosis. J Neurosci.

[19] Clarke LE, Liddelow SA, Chakraborty C, Munch AE, Heiman M, Barres BA (2018). Normal aging induces A1-like astrocyte reactivity. Proc Natl Acad Sci U S A.

[20] Lian H, Yang L, Cole A, Sun L, Chiang AC, Fowler SW (2015). NFkappaB-activated astroglial release of complement C3 compromises neuronal morphology and function associated with Alzheimer’s disease. Neuron.

[21] Anderson MA, Burda JE, Ren Y, Ao Y, O'Shea TM, Kawaguchi R (2016). Astrocyte scar formation aids central nervous system axon regeneration. Nature.

[22] Guttenplan KA, Weigel MK, Adler DI, Couthouis J, Liddelow SA, Gitler AD (2020). Knockout of reactive astrocyte activating factors slows disease progression in an ALS mouse model. Nat Commun.

[23] Shao L, Jiang GT, Yang XL, Zeng ML, Cheng JJ, Kong S (2021). Silencing of circIgf1r plays a protective role in neuronal injury via regulating astrocyte polarization during epilepsy. FASEB J.

[24] King A, Szekely B, Calapkulu E, Ali H, Rios F, Jones S, et al. The increased densities, but different distributions, of both C3 and S100A10 immunopositive astrocyte-like cells in Alzheimer’s disease brains suggest possible roles for both A1 and A2 astrocytes in the disease pathogenesis. Brain Sci. 2020;10(8). 10.3390/brainsci10080503. 10.3390/brainsci10080503 PMC7463428 32751955

[25] Acioglu C, Li L, Elkabes S (2021). Contribution of astrocytes to neuropathology of neurodegenerative diseases. Brain Res.

[26] Kwon HS, Koh SH (2020). Neuroinflammation in neurodegenerative disorders: the roles of microglia and astrocytes. Transl Neurodegener.

[27] Reid JK, Kuipers HF (2021). She doesn’t even go here: the role of inflammatory astrocytes in CNS disorders. Front Cell Neurosci.

[28] Hasel P, Rose IVL, Sadick JS, Kim RD, Liddelow SA (2021). Neuroinflammatory astrocyte subtypes in the mouse brain. Nat Neurosci.

[29] Cavaliere C, Tramontano L, Fiorenza D, Alfano V, Aiello M, Salvatore M (2020). Gliosis and neurodegenerative diseases: the role of PET and MR imaging. Front Cell Neurosci.

[30] Slotkin TA (1999). Mary Bernheim and the discovery of monoamine oxidase. Brain Res Bull.

[31] Manzoor S, Hoda N (2020). A comprehensive review of monoamine oxidase inhibitors as anti-Alzheimer’s disease agents: a review. Eur J Med Chem.

[32] Wang CC, Billett E, Borchert A, Kuhn H, Ufer C (2013). Monoamine oxidases in development. Cell Mol Life Sci.

[33] Fowler JS, Logan J, Shumay E, Alia-Klein N, Wang GJ, Volkow ND (2015). Monoamine oxidase: radiotracer chemistry and human studies. J Label Compd Radiopharm.

[34] Narayanaswami V, Drake LR, Brooks AF, Meyer JH, Houle S, Kilbourn MR (2019). Classics in neuroimaging: development of PET tracers for imaging monoamine oxidases. ACS Chem Neurosci.

[35] Saba W, Valette H, Peyronneau M-A, Bramoullé Y, Coulon C, Curet O (2010). [(11)C]SL25.1188, a new reversible radioligand to study the monoamine oxidase type B with PET: preclinical characterisation in nonhuman primate. Synapse.

[36] Nag S, Fazio P, Lehmann L, Kettschau G, Heinrich T, Thiele A (2016). In vivo and in vitro characterization of a novel MAO-B inhibitor radioligand, 18F-labeled deuterated fluorodeprenyl. J Nucl Med.

[37] Harada R, Hayakawa Y, Ezura M, Lerdsirisuk P, Du Y, Ishikawa Y (2021). (18)F-SMBT-1: a selective and reversible PET tracer for monoamine oxidase-B imaging. J Nucl Med.

[38] Nag S, Lehmann L, Heinrich T, Thiele A, Kettschau G, Nakao R (2011). Synthesis of three novel fluorine-18 labeled analogues of L-deprenyl for positron emission tomography (PET) studies of monoamine oxidase B (MAO-B). J Med Chem.

[39] Ishibashi K, Miura Y, Hirata K, Toyohara J, Ishii K (2020). 18F-THK5351 PET can identify astrogliosis in multiple sclerosis plaques. Clin Nucl Med.

[40] Takami Y, Yamamoto Y, Norikane T, Mitamura K, Hatakeyama T, Nishiyama Y (2020). 18F-THK5351 PET can identify lesions of acute traumatic brain injury. Clin Nucl Med.

[41] Harada R, Ishiki A, Kai H, Sato N, Furukawa K, Furumoto S (2018). Correlations of (18)F-THK5351 PET with postmortem burden of tau and astrogliosis in Alzheimer disease. J Nucl Med.

[42] Murugan NA, Chiotis K, Rodriguez-Vieitez E, Lemoine L, Agren H, Nordberg A (2019). Cross-interaction of tau PET tracers with monoamine oxidase B: evidence from in silico modelling and in vivo imaging. Eur J Nucl Med Mol Imaging.

[43] Li JX (2017). Imidazoline I2 receptors: an update. Pharmacol Ther.

[44] Matthews PM, Datta G (2015). Positron-emission tomography molecular imaging of glia and myelin in drug discovery for multiple sclerosis. Expert Opin Drug Discov.

[45] Kealey S, Turner EM, Husbands SM, Salinas CA, Jakobsen S, Tyacke RJ (2013). Imaging imidazoline-I2 binding sites in porcine brain using 11C-BU99008. J Nucl Med.

[46] Kumar A, Koistinen NA, Malarte ML, Nennesmo I, Ingelsson M, Ghetti B (2021). Astroglial tracer BU99008 detects multiple binding sites in Alzheimer’s disease brain. Mol Psychiatry.

[47] Tournier BB, Tsartsalis S, Ceyzeriat K, Fraser BH, Gregoire MC, Kovari E (2020). Astrocytic TSPO upregulation appears before microglial TSPO in Alzheimer’s disease. J Alzheimers Dis.

[48] Guilarte TR (2019). TSPO in diverse CNS pathologies and psychiatric disease: a critical review and a way forward. Pharmacol Ther.

[49] Boche D, Gerhard A, Rodriguez-Vieitez E, Faculty M (2019). Prospects and challenges of imaging neuroinflammation beyond TSPO in Alzheimer’s disease. Eur J Nucl Med Mol Imaging.

[50] Scholl M, Carter SF, Westman E, Rodriguez-Vieitez E, Almkvist O, Thordardottir S (2015). Early astrocytosis in autosomal dominant Alzheimer’s disease measured in vivo by multi-tracer positron emission tomography. Sci Rep.

[51] Rodriguez-Arellano JJ, Parpura V, Zorec R, Verkhratsky A (2016). Astrocytes in physiological aging and Alzheimer’s disease. Neuroscience.

[52] Smit T, Deshayes NAC, Borchelt DR, Kamphuis W, Middeldorp J, Hol EM (2021). Reactive astrocytes as treatment targets in Alzheimer’s disease-systematic review of studies using the APPswePS1dE9 mouse model. Glia.

[53] Kreisl WC, Kim M-J, Coughlin JM, Henter ID, Owen DR, Innis RB (2020). PET imaging of neuroinflammation in neurological disorders. Lancet Neurol.

[54] Vilaplana E, Rodriguez-Vieitez E, Ferreira D, Montal V, Almkvist O, Wall A (2020). Cortical microstructural correlates of astrocytosis in autosomal-dominant Alzheimer disease. Neurology.

[55] Rodriguez-Vieitez E, Carter SF, Chiotis K, Saint-Aubert L, Leuzy A, Scholl M (2016). Comparison of early-phase 11C-deuterium-l-deprenyl and 11C-Pittsburgh compound B PET for assessing brain perfusion in Alzheimer disease. J Nucl Med.

[56] Carter SF, Scholl M, Almkvist O, Wall A, Engler H, Langstrom B (2012). Evidence for astrocytosis in prodromal Alzheimer disease provided by 11C-deuterium-L-deprenyl: a multitracer PET paradigm combining 11C-Pittsburgh compound B and 18F-FDG. J Nucl Med.

[57] Choo IL, Carter SF, Scholl ML, Nordberg A (2014). Astrocytosis measured by (1)(1)C-deprenyl PET correlates with decrease in gray matter density in the parahippocampus of prodromal Alzheimer’s patients. Eur J Nucl Med Mol Imaging.

[58] Rodriguez-Vieitez E, Ni R, Gulyas B, Toth M, Haggkvist J, Halldin C (2015). Astrocytosis precedes amyloid plaque deposition in Alzheimer APPswe transgenic mouse brain: a correlative positron emission tomography and in vitro imaging study. Eur J Nucl Med Mol Imaging.

[59] Calsolaro V, Matthews PM, Donat CK, Livingston NR, Femminella GD, Guedes SS (2021). Astrocyte reactivity with late-onset cognitive impairment assessed in vivo using (11)C-BU99008 PET and its relationship with amyloid load. Mol Psychiatry.

[60] Nagele RG, D'Andrea MR, Lee H, Venkataraman V, Wang H-Y (2003). Astrocytes accumulate A beta 42 and give rise to astrocytic amyloid plaques in Alzheimer disease brains. Brain Res.

[61] Olabarria M, Noristani HN, Verkhratsky A, Rodríguez JJ (2010). Concomitant astroglial atrophy and astrogliosis in a triple transgenic animal model of Alzheimer’s disease. Glia.

[62] Lee HJ, Lee EC, Seo S, Ko KP, Kang JM, Kim WR (2020). Identification of heterogeneous subtypes of mild cognitive impairment using cluster analyses based on PET imaging of Tau and astrogliosis. Front Aging Neurosci.

[63] Verberk IMW, Thijssen E, Koelewijn J, Mauroo K, Vanbrabant J, de Wilde A (2020). Combination of plasma amyloid beta(1–42/1-40) and glial fibrillary acidic protein strongly associates with cerebral amyloid pathology. Alzheimers Res Ther.

[64] Benedet AL, Mila-Aloma M, Vrillon A, Ashton NJ, Pascoal TA, Lussier F (2021). Differences between plasma and cerebrospinal fluid glial fibrillary acidic protein levels across the Alzheimer disease continuum. JAMA Neurol.

[65] Pereira JB, Janelidze S, Smith R, Mattsson-Carlgren N, Palmqvist S, Teunissen CE (2021). Plasma GFAP is an early marker of amyloid-beta but not tau pathology in Alzheimer’s disease. Brain.

[66] Verberk IMW, Slot RE, Verfaillie SCJ, Heijst H, Prins ND, van Berckel BNM (2018). Plasma amyloid as prescreener for the earliest Alzheimer pathological changes. Ann Neurol.

[67] Alcolea D, Martínez-Lage P, Sánchez-Juan P, Olazarán J, Antúnez C, Izagirre A (2015). Amyloid precursor protein metabolism and inflammation markers in preclinical Alzheimer disease. Neurology.

[68] Mila-Aloma M, Salvado G, Gispert JD, Vilor-Tejedor N, Grau-Rivera O, Sala-Vila A (2020). Amyloid beta, tau, synaptic, neurodegeneration, and glial biomarkers in the preclinical stage of the Alzheimer’s continuum. Alzheimers Dement.

[69] Aarsland D, Batzu L, Halliday GM, Geurtsen GJ, Ballard C, Ray Chaudhuri K (2021). Parkinson disease-associated cognitive impairment. Nat Rev Dis Primers.

[70] Halliday GM, Stevens CH. Glia: initiators and progressors of pathology in Parkinson’s disease. Mov Disord. 2011;26(1). 10.1002/mds.23455. 10.1002/mds.23455 21322014

[71] di Domenico A, Carola G, Calatayud C, Pons-Espinal M, Muñoz JP, Richaud-Patin Y (2019). Patient-specific iPSC-derived astrocytes contribute to non-cell-autonomous neurodegeneration in Parkinson’s disease. Stem Cell Rep.

[72] Parker CA, Nabulsi N, Holden D, Lin S-f, Cass T, Labaree D (2014). Evaluation of 11C-BU99008, a PET ligand for the imidazoline2 binding sites in rhesus brain. J Nucl Med.

[73] Tyacke RJ, Myers JFM, Venkataraman A, Mick I, Turton S, Passchier J (2018). Evaluation of C-BU99008, a PET ligand for the imidazoline binding site in human brain. J Nucl Med.

[74] Tong J, Rathitharan G, Meyer JH, Furukawa Y, Ang L-C, Boileau I (2017). Brain monoamine oxidase B and A in human parkinsonian dopamine deficiency disorders. Brain.

[75] Tong J, Ang LC, Williams B, Furukawa Y, Fitzmaurice P, Guttman M (2015). Low levels of astroglial markers in Parkinson’s disease: relationship to alpha-synuclein accumulation. Neurobiol Dis.

[76] Schonecker S, Brendel M, Palleis C, Beyer L, Hoglinger GU, Schuh E (2019). PET imaging of astrogliosis and tau facilitates diagnosis of Parkinsonian syndromes. Front Aging Neurosci.

[77] Van Harten ACM, Phatnani H, Przedborski S (2021). Non-cell-autonomous pathogenic mechanisms in amyotrophic lateral sclerosis. Trends Neurosci.

[78] Vahsen BF, Gray E, Thompson AG, Ansorge O, Anthony DC, Cowley SA (2021). Non-neuronal cells in amyotrophic lateral sclerosis - from pathogenesis to biomarkers. Nat Rev Neurol.

[79] Tam OH, Rozhkov NV, Shaw R, Kim D, Hubbard I, Fennessey S, et al. Postmortem cortex samples identify distinct molecular subtypes of ALS: retrotransposon activation, oxidative stress, and activated glia. Cell Rep. 2019;29(5). 10.1016/j.celrep.2019.09.066. 10.1016/j.celrep.2019.09.066 PMC6866666 31665631

[80] Schiffer D, Cordera S, Cavalla P, Migheli A (1996). Reactive astrogliosis of the spinal cord in amyotrophic lateral sclerosis. J Neurol Sci.

[81] Nagy D, Kato T, Kushner PD (1994). Reactive astrocytes are widespread in the cortical gray matter of amyotrophic lateral sclerosis. J Neurosci Res.

[82] Vargas MR, Pehar M, Díaz-Amarilla PJ, Beckman JS, Barbeito L (2008). Transcriptional profile of primary astrocytes expressing ALS-linked mutant SOD1. J Neurosci Res.

[83] Johansson A, Engler H, Blomquist G, Scott B, Wall A, Aquilonius S-M (2007). Evidence for astrocytosis in ALS demonstrated by [11C](l)-deprenyl-D2 PET. J Neurol Sci.

[84] Kushner PD, Stephenson DT, Wright S (1991). Reactive astrogliosis is widespread in the subcortical white matter of amyotrophic lateral sclerosis brain. J Neuropathol Exp Neurol.

[85] Higashihara M, Ishibashi K, Tokumaru AM, Iwata A, Ishii K (2021). 18F-THK5351 PET can identify core lesions in different amyotrophic lateral sclerosis phenotypes. Clin Nucl Med.

[86] Frohman EM, Racke MK, Raine CS (2006). Multiple sclerosis–the plaque and its pathogenesis. N Engl J Med.

[87] Makhani N, Tremlett H (2021). The multiple sclerosis prodrome. Nat Rev Neurol.

[88] Brosnan CF, Raine CS (2013). The astrocyte in multiple sclerosis revisited. Glia.

[89] Brambilla R (2019). The contribution of astrocytes to the neuroinflammatory response in multiple sclerosis and experimental autoimmune encephalomyelitis. Acta Neuropathol.

[90] Bodini B, Tonietto M, Airas L, Stankoff B (2021). Positron emission tomography in multiple sclerosis - straight to the target. Nat Rev Neurol.

[91] Waniewski RA, Martin DL (1998). Preferential utilization of acetate by astrocytes is attributable to transport. J Neurosci.

[92] Takata K, Kato H, Shimosegawa E, Okuno T, Koda T, Sugimoto T (2014). 11C-acetate PET imaging in patients with multiple sclerosis. PLoS ONE.

[93] Kato H, Okuno T, Isohashi K, Koda T, Shimizu M, Mochizuki H (2021). Astrocyte metabolism in multiple sclerosis investigated by 1-C-11 acetate PET. J Cereb Blood Flow Metab.

[94] Ardaya M, Joya A, Padro D, Plaza-Garcia S, Gomez-Vallejo V, Sanchez M (2020). In vivo PET imaging of gliogenesis after cerebral ischemia in rats. Front Neurosci.

[95] Choudhury GR, Ding S (2016). Reactive astrocytes and therapeutic potential in focal ischemic stroke. Neurobiol Dis.

[96] Rakers C, Schleif M, Blank N, Matuskova H, Ulas T, Handler K (2019). Stroke target identification guided by astrocyte transcriptome analysis. Glia.

[97] Liddelow SA, Guttenplan KA, Clarke LE, Bennett FC, Bohlen CJ, Schirmer L (2017). Neurotoxic reactive astrocytes are induced by activated microglia. Nature.

[98] Chen M-K, Guilarte TR (2006). Imaging the peripheral benzodiazepine receptor response in central nervous system demyelination and remyelination. Toxicol Sci.

[99] Chen M-K, Guilarte TR. Translocator protein 18 kDa (TSPO): molecular sensor of brain injury and repair. Pharmacol Ther. 2008;118(1). 10.1016/j.pharmthera.2007.12.004. 10.1016/j.pharmthera.2007.12.004 PMC2453598 18374421

[100] Jarius S, Paul F, Weinshenker BG, Levy M, Kim HJ, Wildemann B (2020). Neuromyelitis optica. Nat Rev Dis Primers.

[101] Guo Y, Lennon VA, Parisi JE, Popescu B, Vasquez C, Pittock SJ (2021). Spectrum of sublytic astrocytopathy in neuromyelitis optica. Brain.

[102] Higashiyama A, Komori T, Inada Y, Nishizawa M, Nakajima H, Narumi Y (2017). Diffuse (18)F-FDG uptake throughout the spinal cord in the acute phase of Neuromyelitis Optica Spectrum disorder. Eur J Nucl Med Mol Imaging.

[103] Lopez-Mora DA, Flotats A, Fernandez A, Sizova M, Camacho V, Carrio I (2020). Striking neurologic 18F-FDG PET/CT pattern in Devic’s disease (neuromyelitis optica spectrum disorder). Eur J Nucl Med Mol Imaging.

[104] Kimura A, Takekoshi A, Yoshikura N, Hayashi Y, Shimohata T (2019). Clinical characteristics of autoimmune GFAP astrocytopathy. J Neuroimmunol.

[105] Sosunov A, Olabarria M, Goldman JE (2018). Alexander disease: an astrocytopathy that produces a leukodystrophy. Brain Pathol.

